# DHX15 Inhibits Autophagy and the Proliferation of Hepatoma Cells

**DOI:** 10.3389/fmed.2020.591736

**Published:** 2021-02-11

**Authors:** Miaomiao Zhao, Lixiong Ying, Rusha Wang, Jiping Yao, Liming Zhu, Min Zheng, Zhi Chen, Zhenggang Yang

**Affiliations:** ^1^The State Key Laboratory for Diagnosis and Treatment of Infectious Diseases, College of Medicine, The First Affiliated Hospital, Zhejiang University, Hangzhou, China; ^2^Collaborative Innovation Center for Diagnosis and Treatment of Infectious Diseases, Hangzhou, China; ^3^Pathology Department, College of Medicine, The First Affiliated Hospital, Zhejiang University, Hangzhou, China; ^4^Department of Chemotherapy, Zhejiang Cancer Hospital, Hangzhou, China

**Keywords:** DEAH-box helicase 15, RNA helicase, autophagy, mTORC1, HCC cells

## Abstract

Autophagy is a highly conserved process by which superfluous or harmful components in eukaryotic cells are degraded by autophagosomes. This cytoprotective mechanism is strongly related to various human diseases, such as cancer, autoimmune diseases, and diabetes. DEAH-box helicase 15 (DHX15), a member of the DEAH box family, is mainly involved in RNA splicing and ribosome maturation. Recently, DHX15 was identified as a tumor-related factor. Although both autophagy and DHX15 are involved in cellular metabolism and cancer progression, their exact relationship and mechanism remain elusive. In this study, we discovered a non-classic function of DHX15 and identified DHX15 as a suppressive protein in autophagy for the first time. We further found that mTORC1 is involved in DHX15-mediated regulation of autophagy and that DHX15 inhibits proliferation of hepatocellular carcinoma (HCC) cells by suppressing autophagy. In conclusion, our study demonstrates a non-classical function of DHX15 as a negative regulator of autophagy related to the mTORC1 pathway and reveals that DHX15-related autophagy dysfunction promotes HCC cell proliferation, indicating that DHX15 may be a target for liver cancer treatment.

## Introduction

Autophagy is a highly conserved mechanism by which redundant cellular components are degraded in double-membrane vesicles called autophagosomes. During this process, the outer membrane of a autophagosome fuses with a lysosome, after which acidic hydrolases degrade autophagic cargo and subsequently recycle the macromolecules (1, 2). Under physiological conditions, basal autophagy helps cells discard damaged organelles, degrade misfolded proteins, resist exogenous microorganisms, and maintain genomic stability, thus protecting the body from chronic damage and inflammation and inhibiting cancer initiation (3, 4). However, under abnormal conditions, long-term, excessive or defective autophagy is harmful and is closely related to the occurrence and development of cancers, autoimmune diseases, heart failure, neurodegenerative diseases and other diseases (5–8). In the case of cancer, autophagy can be used by cancer cells to defend against host immune attack, thus enhancing cancer cell survival, invasion and metastasis (9–11). Therefore, precise regulation of autophagy is extremely important for the maintenance of cellular homeostasis and treatment of several diseases.

DEAH-box 15 (DHX15), an important member of the RNA helicase family, is involved mainly in pre-mRNA splicing and promotion of ribosome maturation (12–14). Most reported studies have concentrated on its splicing function in RNA metabolism (15–17). However, DHX15 exhibits various functions in human tumorigenesis through transcriptional or post-transcriptional regulation independent of its ATPase activity (18, 19) or through interaction with proteins that have a G-patch domain (20). DHX15 enhances androgen receptor (AR) transcriptional activity by stimulating Siah2-mediated ubiquitination, which contributes to the progression of prostate cancer (19). In glioma, DHX15 is an antitumour gene, and its Ia–Ib and III–IV motifs, but not its ATPase activity, play important roles in its growth-inhibitory function (18). Moreover, DHX15 not only dysregulates genes downstream of the NF-kB signaling pathway, an important mechanism of its antitumour function, but also downregulates genes involved in splicing and ribosomal biogenesis in glioma, indicating that DHX15 regulates glioma at the post-transcriptional level (18). The G-patch is a glycine-rich domain that interacts with DEAH helicases to enhance their activity (21–23). For example, the G-patch domain of the GPATCH2 protein interacts with DHX15, which promotes breast cancer cell growth (20). Therefore, DHX15 is a versatile protein that participates in different stages of many types of cancers. In addition, DHX15 has been shown to aid in the diagnosis of acute lymphoblastic leukemia (AML) (24, 25) and to act as a viral RNA sensor in antiviral responses and innate immunity through the MAVS-mediated signaling pathway and the Nlrp6-interferon pathway (26, 27). Recently, DHX15 has been shown to be associated with clinically pathological elements and prognosis in patients with hepatocellular carcinoma (HCC) (28).

Autophagy is a highly conserved mechanism in cells proven to be closely related to the occurrence and progression of tumors. DHX15, a member of RNA helicase family, is also highly conserved in structure and function. Recently, DHX15 has been reported to be a tumor-related factor. However, the relationship between autophagy and DHX15 remains unclear. Nor is it clear whether this relationship plays a role in cancer. In this study, we found the relationship between autophagy and DHX15 and identified DHX15 as an autophagy suppressor for the first time. Knockdown of endogenous DHX15 dramatically induced autophagy independent of the function of DHX15 in regulating RNA metabolism. In addition, downregulation of DHX15 inhibited activation of mTORC1. Furthermore, we found that DHX15 acts as a suppressor of proliferation in hepatoma cells and that this function is dependent on autophagy inhibition. Collectively, these results show that DHX15 inhibits autophagy by activating mTORC1, which is associated with the anti-tumor effect of DHX15.

## Results

### Downregulation of Endogenous DHX15 Induces Autophagy

**Figure 1:**
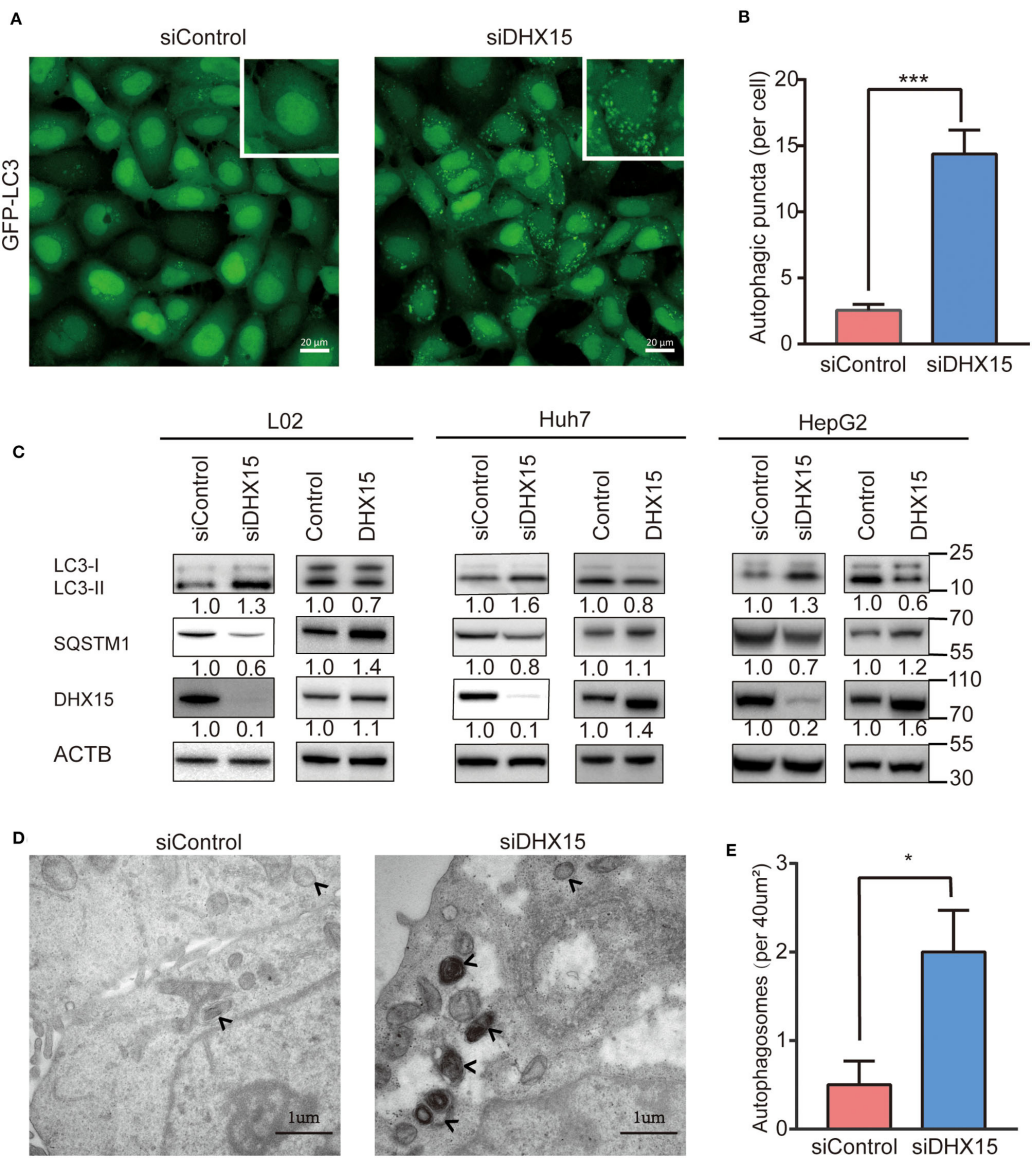
Knockdown of DHX15 induces autophagy. **(A)** GFP-LC3 puncta were analyzed in HEK293 cells stably expressing GFP-LC3 (293-GFP-LC3 cells) after transient transfection with DHX15 siRNA (siDHX15) or a scrambled control (siControl). Representative images were obtained at 600× magnification. Scale bars: 20 μm. **(B)** The number of GFP-LC3 puncta per cell was quantified for each group. The data are presented as the mean ± SEM. Unpaired Student's *t-*test (*n* = 5). ***P < 0.001. **(C)** LC3-II conversion and SQSTM/p62 degradation in L02, Huh7, and HepG2 cells that had been transiently transfected with siDHX15 or siControl were detected by western blotting. LC3-II conversion and SQSTM1/p62 degradation in these three cell lines that had been transiently transfected by DHX15 plasmid or control plasmid were detected by western blotting. *P < 0.05. **(D)** Autophagosomes in L02 cells were analyzed by transmission electron microscopy. The autophagosomes are indicated by arrows. Scale bars: 1 μm. **(E)** Numbers of autophagosomes per 40 μm^2^ as determined from the images. The data are presented as the mean ± SEM. Unpaired Student's *t-*test (*n* = 10). *P < 0.05.

To examine the effect of DHX15 on autophagy, we temporarily knocked down endogenous DHX15 in different human cell lines after transfection with small interfering RNA (siRNA) and detected the protein expression of microtubule-associated protein 1 light chain 3 (LC3) and SQSTM/p62, important biomarkers of autophagy. As shown in Figure 1A, we first visualized autophagic activity in 293-GFP-LC3 cells (HEK293 cells with stable expression of GFP-LC3) by fluorescence microscopy. Compared with control cells, 293-GFP-LC3 cells in which endogenous DHX15 expression was knocked down exhibited significantly more GFP-LC3 puncta (Figures 1A,B). Similarly, DHX15 silencing in both L02 hepatocytes and HepG2 and Huh7 HCC cells significantly promoted LC3-II conversion and SQSTM1/P62 degradation (Figure 1C and Supplementary Figure 1). In addition to detecting the effect of DHX15 knockdown, we found that overexpression of DHX15 in these cell lines suppressed autophagy under basal conditions (Figure 1C and Supplementary Figure 1; the data in Supplementary Figure 1 were obtained from three independent experiments). Data are presented in Supplementary Figure 1 obtained from three independent experiments. To further confirm this function, autophagosomes were quantified by transmission electron microscopy. Downregulation of DHX15 significantly increased the number of autophagosomes in L02 cells (Figures 1D,E). Taken together, these data suggest that downregulation of endogenous DHX15 dramatically induces autophagy and indicate that DHX15 is a suppressor of autophagy.

### Knockdown of DHX15 Promotes the Formation of Autophagosomes

**Figure 2:**
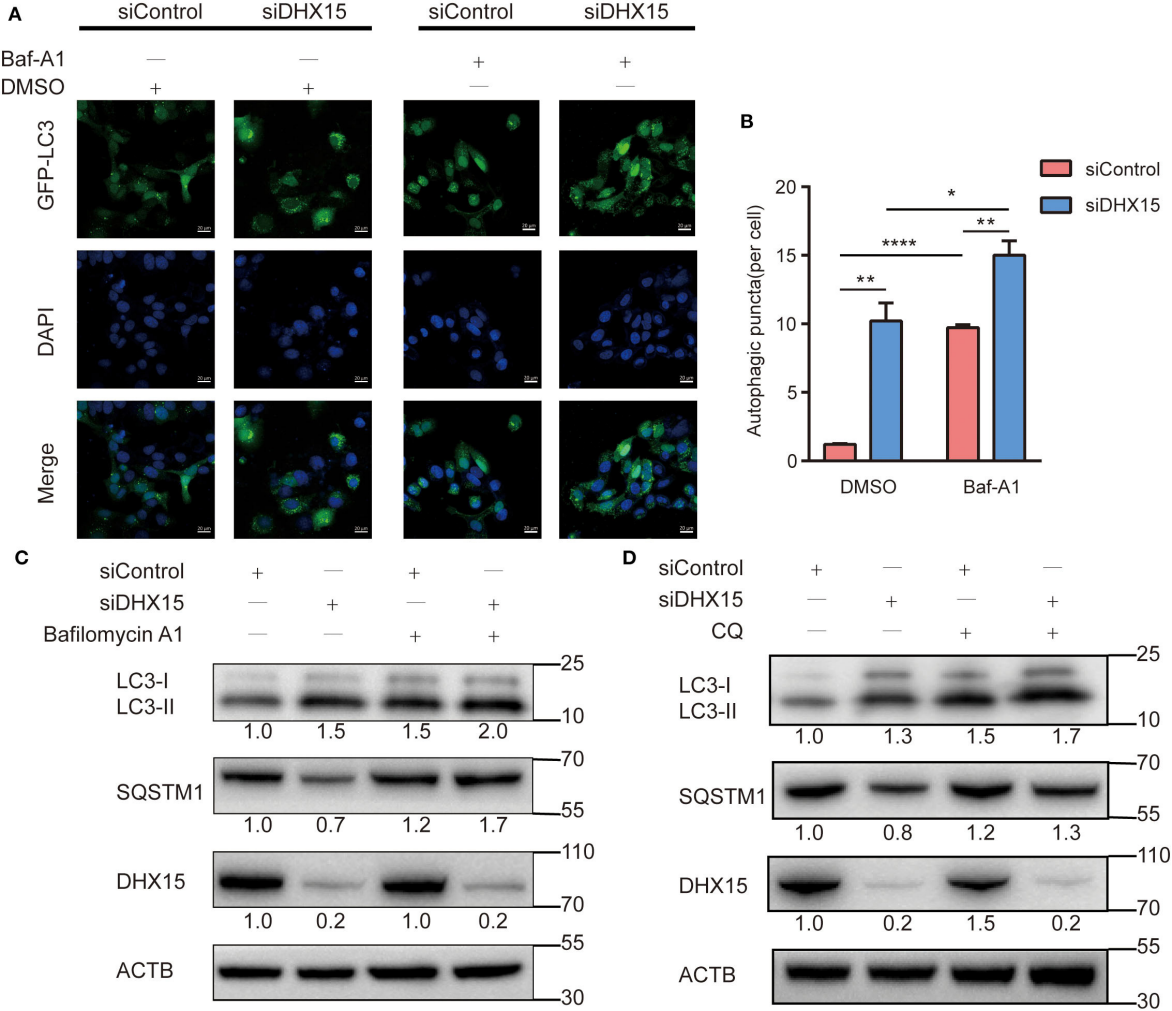
Depletion of DHX15 stimulates autophagosome generation. **(A)** GFP-LC3 puncta were analyzed in Huh7 cells transiently cotransfected with DHX15 siRNA (siDHX15) or scrambled control siRNA (siControl) and GFP-LC3 plasmids with or without Baf-A1 (200 nM) treatment for 6 h. Cellular nuclei were counterstained with DAPI. Representative images were obtained at 600× magnification. Scale bars: 20 μm. **(B)** The number of GFP-LC3 puncta per cell was quantified for each group. The data are presented as the mean ± SEM. Unpaired Student's *t-*test (*n* = 5). *P < 0.05, **P < 0.01, ****P < 0.0001. **(C)** The expression of SQSTM1/p62 and LC3B-II in Huh7 cells transiently transfected with siDHX15 or siControl with or without Baf-A1 (200 nM) treatment for 6 h was measured by western blotting. *P < 0.05. **(D)** The expression of SQSTM1/p62 and LC3B-II in Huh7 cells transiently transfected with siDHX15 or siControl with or without chloroquine diphosphate(CQ) (20 uM) treatment for 24 h were measured by western blotting.

Autophagic flux is responsible for the dynamic nature of autophagy and consists of 3 sequential steps: autophagosome generation, autolysosome formation and degradation (29). We hypothesized that increased autophagosome formation and/or decreased autophagic degradation may have been the cause of the DHX15 knockdown-induced GFP-LC3 puncta generation. To determine whether autophagic flux is affected by DHX15, we treated cells with the late-stage autophagy inhibitor bafilomycin A1 (Baf-A1), a vacuolar-type H^+^-translocating ATPase inhibitor, after DHX15 silencing. We found that the conversion of LC3-II and the number of GFP-LC3 puncta were significantly augmented by Baf-A1 treatment in cells with knockdown of endogenous DHX15, indicating that DHX15 regulates autophagy by increasing autophagosome formation. Figure 2A shows that knockdown of DHX15 dramatically augmented GFP-LC3 puncta formation after treatment with Baf-A1 (Figures 2A,B). We also found that the conversion of LC3-II was increased by Baf-A1 treatment after downregulation of DHX15 (Figure 2C). As shown in Figure 2D, consistent with these findings, SQSTM1/p62 degradation was more obvious in DHX15-knockdown cells than in control cells, and the DHX15 knockdown-induced degradation was inhibit by Baf-A1 treatment (Figure 2C and Supplementary Figure 2). The similar results also show in chloroquine diphosphate(CQ) treatment (Figure 2D and Supplementary Figure 2). Collectively, these results indicate that knockdown of DHX15 augments the formation of autophagosomes but does not decrease autophagic degradation, revealing that DHX15 functions in an early step before autophagosome fusion.

### DHX15 Regulates Autophagy in a Manner Associated With mTORC1 Activation

**Figure 3:**
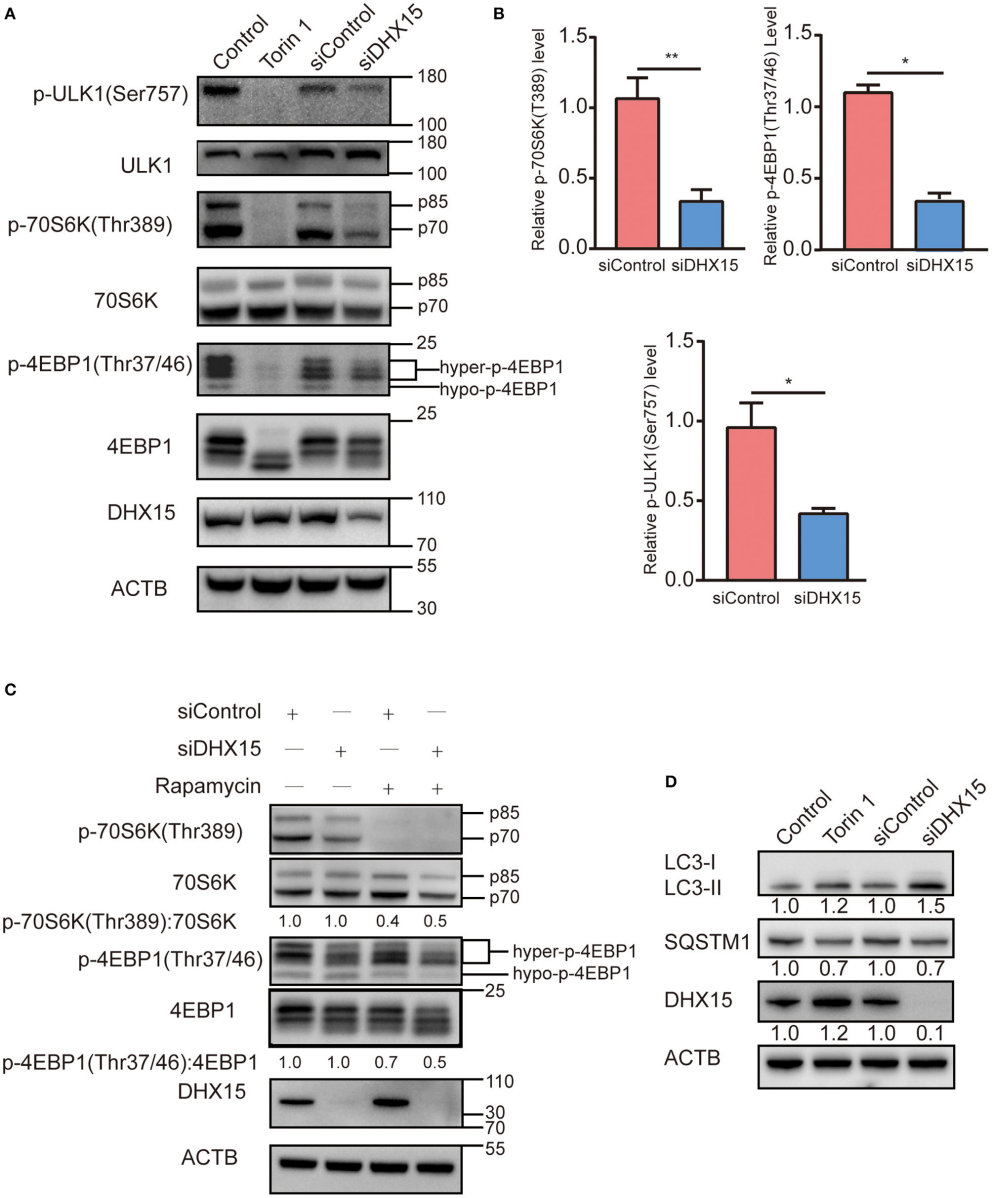
Depletion of DHX15 induces autophagy associated with suppressing mTORC1. **(A)** The levels of phosphorylated substrates of the mTORC1 complex in HepG2 cells transiently transfected with DHX15 siRNA (siDHX15) or scrambled control siRNA (siControl) were analyzed by western blotting. Treatment with Torin 1 (250 nM) for 6 h was used as a positive control. Treatment with DMSO was used as a negative control. β-Actin was used as a loading control. *P < 0.05. **(B)** Data are representative western blots of three independent assays. *P < 0.05, **P < 0.001 **(C)** HepG2 cells transiently transfected with siDHX15 or siControl were treated with 200 nM rapamycin for 2 h and harvested for western blotting. Treatment with DMSO was used as a negative control. β-Actin was used as a loading control. *P < 0.05. **(D)** LC3 and SQSTM1/p62 levels in HepG2 cells transiently transfected with siDHX15 or siControl were measured by western blotting. Treatment with Torin 1 (250 nM) for 6 h was used as a positive control. *P < 0.05.

MTORC1 is a key upstream inhibitor of autophagy that balances autophagy and other cellular physiological processes, such as cell growth (30). Since DHX15 is also an inhibitor of autophagy, we hypothesized that mTORC1 and DHX15 work together to achieve autophagy regulation. To test this hypothesis, we investigated the phosphorylation statuses of RPS6KB/p70S6K, initiation factor 4E binding protein 1 (4EBP1) and ULK1, which are signaling molecules downstream of mTORC1, after endogenous DHX15 knockdown. We found that p70S6K and p4EBP1 protein levels dramatically declined when DHX15 was knocked down (Figure 3A). We further measured the level of ULK1 phosphorylation at residue Ser757, which negatively affects autophagy initiation by disrupting the interaction of AMPK and ULK1 mediated by mTORC1 (31). Consistent with the initial results, our data showed that the level of ULK1 phosphorylation at Ser757 was decreased by DHX15 depletion (Figure 3A). Data quantification of western blots are shown in Figure 3B and representative western blots of three independent assays. Autophagy was induced upon Torin1 treatment or siDHX15 treatment (Figure 3D and Supplementary Figure 3).

Rapamycin is a classic inhibitor of mTOR, and previous research has shown that this inhibitor inhibits growth and angiogenesis in metastatic tumors by reducing VEGF production and blocking VEGF-induced endothelial cell signaling in a CT-26 cell-transplanted tumor model (32). We found that downregulation of DHX15 promoted degradation of both phosphorylation and dephosphorylation substrates of mTORC1 upon rapamycin treatment (Figure 3C). In addition, we also examined the autophagy level after siDHX15 and rapamycin treatment. The results showed that depletion of DHX15 promoted rapamycin-induced autophagy (Supplementary Figure 3).

These data indicate that DHX15 regulates autophagy involved in mTORC1 pathway. Therefore, DHX15 inhibits autophagy in a manner associated with mTORC1 activation.

### DHX15 Inhibits the Proliferation of Hepatoma Cells in an Autophagy-Dependent Manner

**Figure 4:**
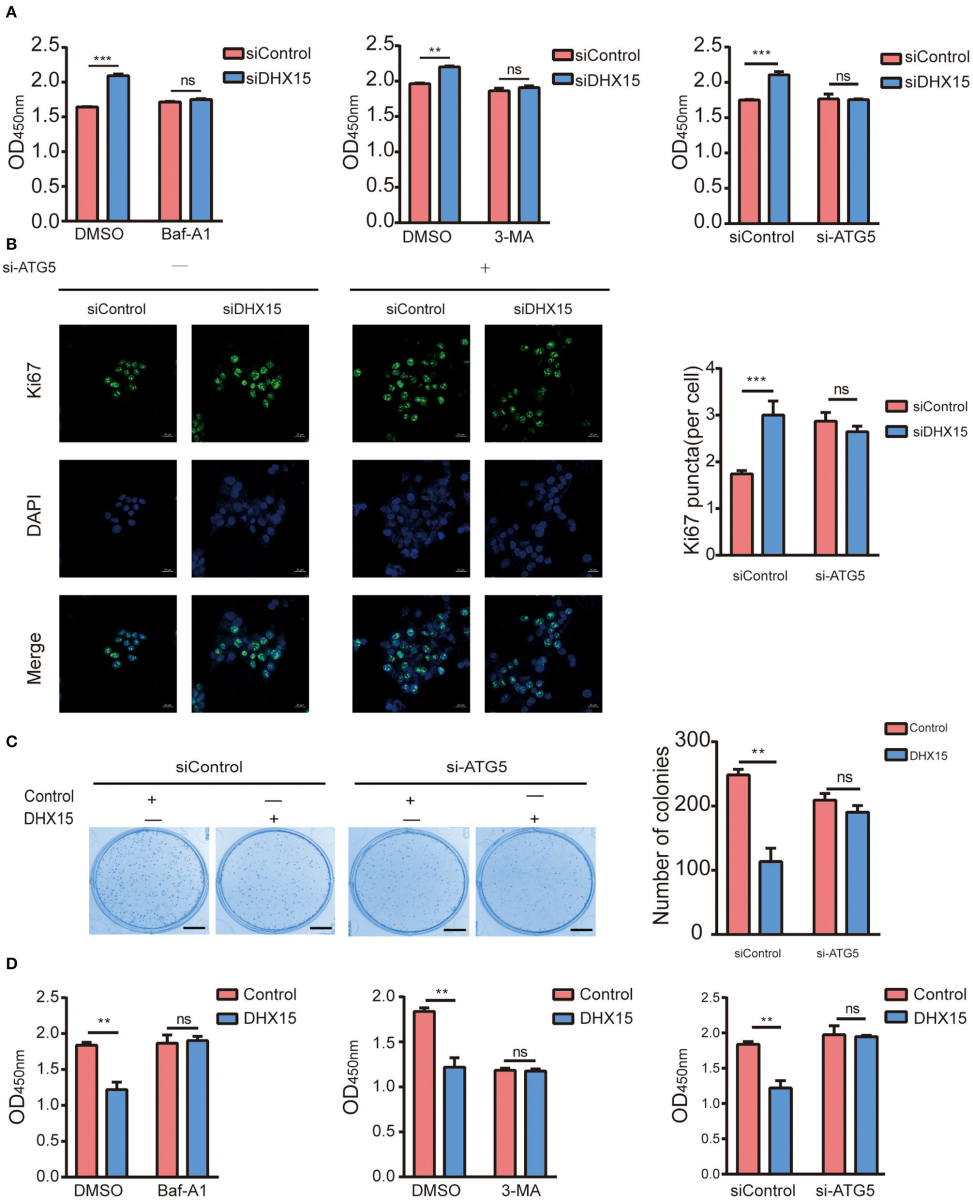
DHX15 suppresses the growth of HCC cells by inhibiting autophagy. **(A)** The relative viability of DHX15-knockdown cells with or without autophagy inhibition was measured by CCK-8 assay. The data are presented as the mean ± SEM (*n* = 3). ***P < 0.001. **(B)** The Ki67-positive puncta in DHX15-knockdown HepG2 cells with or without ATG5 siRNA (si-ATG5) treatment were analyzed by immunofluorescence. Cellular nuclei were counterstained with DAPI. Representative images were obtained at 600× magnification. Scale bars: 20 μm. The graph shows the results of statistical analysis of the numbers of Ki67-positive puncta per cell. The data are presented as the mean ± SEM (*n* = 10). ***P < 0.001. **(C)** After HepG2 cells had been cotransfected with or without DHX15 and si-ATG5 and incubated for 12 days, colonies were stained with Coomassie brilliant blue and counted (scale bar: 5 mm). The data are the mean ± SEM (*n* = 3). **P < 0.01. **(D)** The relative viability of cells overexpressing DHX15 with or without autophagy inhibition was measured by CCK-8 assay. The data are presented as the mean ± SEM (*n* = 3). **P < 0.01.

RNA helicases have been demonstrated to play vital roles in the growth, invasion, and metastasis of HCC (33, 34) and can be used to closely evaluate the prognoses of HCC patients (35). In recent years, DHX15 has been identified as a crucial tumor related protein (18, 19, 28, 36). In addition, the expression status of DHX15 in HCC patients has been described (28). However, the molecular mechanism by which DHX15 regulates HCC is elusive. To determine whether DHX15 is related to HCC, we knocked down DHX15, which markedly increased the growth of HepG2 cells (Figure 4A). Consistent with this finding, DHX15 knockdown also increased the number of puncta positive for Ki67 (a biomarker of cell proliferation) in HepG2 cells (Figure 4B). To further confirm this effect, DHX15 plasmid DNA was transfected into HepG2 cells, and the data showed that overexpression of DHX15 attenuated the proliferation and colony formation of HepG2 cells (Figures 4C,D).

**Figure 5:**
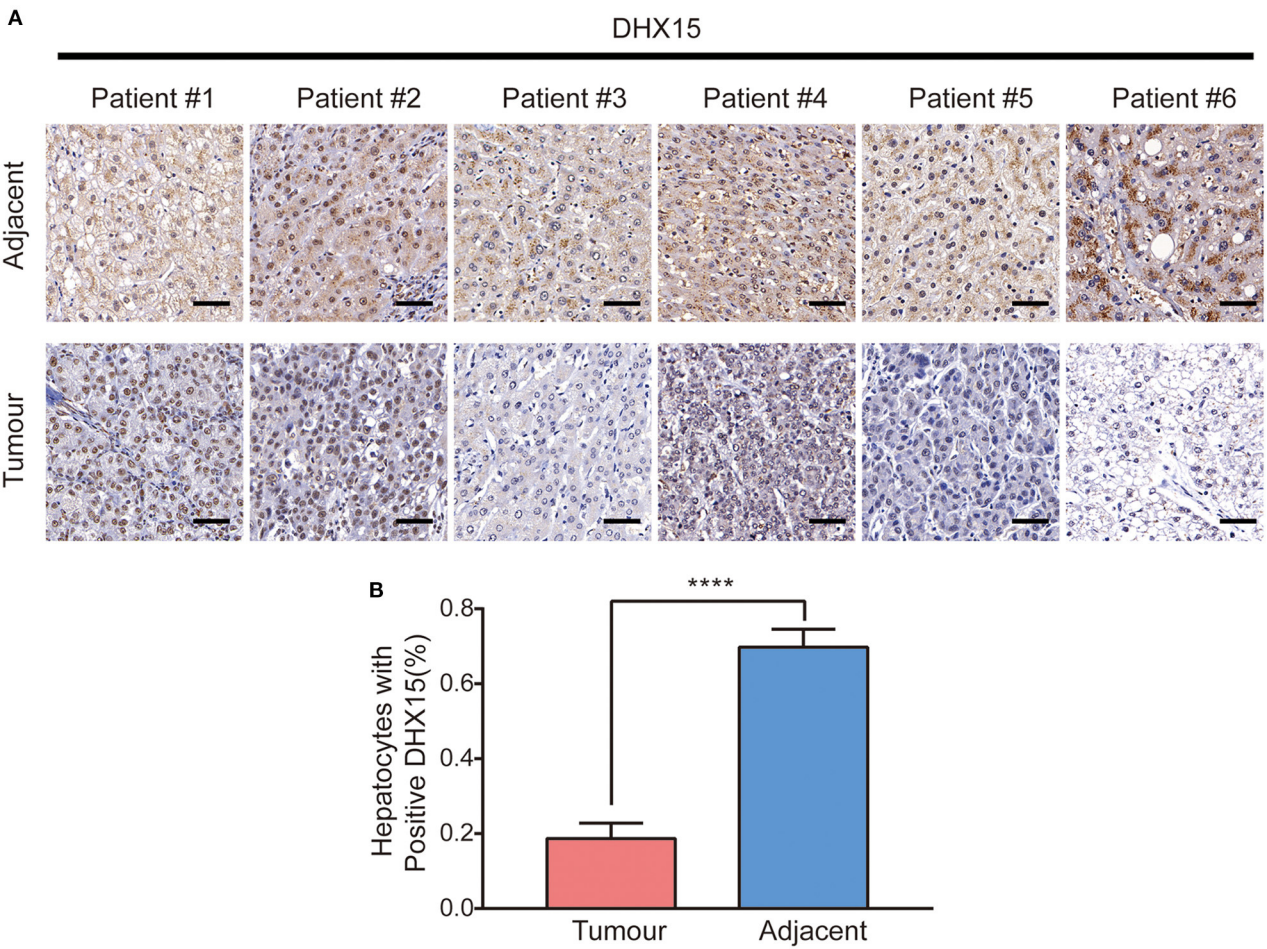
Expression of DHX15 in human liver cancer tissues. **(A)** Expression of DHX15 was detected with immunohistologic staining in tumor tissues and adjacent non-tumor tissues of the representative liver cancer patients. Scale bars: 50 um. **(B)** Plots of percentage of DHX15 positive cells to total cells in human liver cancer tissues. The data are presented as the mean ± SEM. Unpaired Student's *t-*test (*n* = 20). ****P < 0.0001.

Some studies have demonstrated that autophagy levels are low in HCC (37, 38). However, recent studies have also revealed that autophagy plays contrasting roles in HCC and hepatoma cells (10, 39, 40). We found that DHX15 is a novel suppressor of autophagy. To determine whether autophagy participates in the process by which DHX15 regulates HCC, we treated cells with Baf-A1 (a late-stage autophagy inhibitor) and 3-methyladenine (3-MA, an early-stage autophagy inhibitor). Depletion of DHX15 promoted HCC cell proliferation, but this effect disappeared after treatment with the inhibitors (Figures 4A,B). In addition, we used Supplementary Figure 5 verified the ATG5 siRNA is effective in HepG2 cells to further verify this effect and obtained a consistent result (Figures 4A,B). Moreover, overexpression of DHX15 inhibited HepG2 cell growth; this effect was also inhibited by autophagy inhibitor treatment and ATG5 siRNA transfection (Figures 4C,D). In addition, we also detect the expression of DHX15 in human liver cancer tissues. The data showed that the levels of DHX15 (mainly distributed in the cytoplasm) were higher in adjacent normal tissues than in tumor tissues (15 of the 20 sample pairs, Figures 5A,B). These findings indicate that DHX15 inhibits the proliferation of HCC cells in an autophagy-dependent manner.

## Discussion

In the present study, we found, for the first time, that the RNA helicase DHX15 is a negative regulator of autophagy and that MTORC1 is involved in the process by which DHX15 regulates autophagy. Moreover, we found that DHX15 silencing prominently increases HCC cell proliferation and that this effect can be suppressed by autophagy inhibition.

The DEAD/H box helicase family includes various members, and emerging studies have reported the mechanisms of DEAD/H box helicases in autophagy. For example, a recent study demonstrated that DDX5 positively regulates autophagy by interacting with p62 to suppress HCC tumorigenesis (41). The authors also found that DDX5 disrupts the binding of p62 and TRAF6 (a tumor-related protein) and inhibits the activation of mTORC1 (41). Another study has revealed that DDX6 negatively regulates autophagy (6). DDX6 has been shown to bind the decapping enzyme Dcp2, leading to the degradation of autophagic gene mRNA (6). In this study, DHX15 suppressed autophagy in a manner associated with the activation of mTORC1; this mechanism is different from the mechanism by which DDX5 regulates autophagy. We also found that DHX15 did not influence autophagic gene mRNA levels. Consistent with previous work (6), our study revealed that downregulation of DDX6 in HepG2 cells enhanced the mRNA expression of autophagy genes (Supplementary Figure 4); however, the mRNA expression profile in our study differed from that in the previous work, possibly because of the different species and cell lines used. These results indicate that DHX15 inhibits autophagy in other ways, such as by regulating other mRNA molecules or by interacting with other proteins such as G-patch proteins. Therefore, we have identified DHX15 as a novel inhibitor of autophagy that associates with mTORC1 and does not affect the mRNA expression of autophagy genes.

Autophagy dysfunction leads to many human pathologies, such as neurodegenerative diseases (8), liver injury (42) and cancer (37). Basal autophagy maintains genomic stability, protects the body from chronic diseases, and inhibits excessive inflammation under physiological conditions (4). Under pathological conditions, however, autophagy helps cancer cells resist host immune attack or other stressful conditions (9–11). Therefore, precise autophagy regulation is crucial. Autophagy inhibition, which takes place under many conditions, such as viral infection (43), post-prandial lipid assimilation in hepatic cells (44), and neurodegenerative diseases and cancer (45), is an essential part of autophagy regulation. In this study, DHX15 was identified as an autophagy suppressor. MTORC1 is the most important upstream suppressor of autophagy, and we found that DHX15 negatively regulates autophagy in a manner associated with mTORC1 activation (Figure 3). More importantly, we found that depletion of DHX15 increased hepatoma cell proliferation in a manner dependent on the DHX15-mediated regulation of autophagy. These results indicate the existence of a relationship between DHX15 and autophagy inhibition that is related to the antitumour effect of DHX15.

Autophagy is a key process in the maintenance of cellular homeostasis. Factors that disrupt autophagy balance can lead to many human diseases, such as cancer. However, autophagy is a double-edged sword, and its role in cancer is complicated (46). Autophagy participates in many types of cancers but exerts completely opposite effects on tumor progression (47, 48). The dynamic and complex conditions under which cancer cells survive are determined by the microenvironment. Although much about the relationship between autophagy and cancer remains unknown, it is clear that restoration of aberrant autophagy is a new target for disease treatment. In general, DHX15 is thought to regulate RNA metabolism. We found that depletion of DHX15 markedly induced autophagy, which suggests that DHX15 plays an essential role in maintaining the homeostasis of the cellular environment that is independent of its classical function in regulating RNA metabolism. We also detected the expression of DHX15 in human liver tissues (Figure 5A) which suggested DHX15 may be a vital factor in liver cancer. Overall, this study has identified a non-classic function of the RNA helicase DHX15 as a suppressor of autophagy and liver cancer, and the findings suggest that DHX15 may be a potential therapeutic element for liver cancer and autophagy-related diseases. And the mechanism between DHX15 and autophagy maybe the potential prognostic indicator in cancer therapy. In contrast, DHX15 may be a new clinical indicator of liver cancer progression and outcome. However, the precise mechanism of the antitumour effect of DHX15 is not further discussed in this study. Moreover, identification of the precise target by which DHX15 regulates autophagy, which may contribute to further exploration of the role of DHX15 in liver cancer, remains to be identified. In addition, although we have revealed the role of DHX15 in some human liver cancer tissues, an examination of more samples is required to fully demonstrate its role, and the clinic outcome of liver cancer patients should be examined.

## Materials and Methods

### Cell Culture and Treatments

Hepatocyte lines (Huh7 and HepG2) and HEK293 cells with stable LC3-GFP expression were cultured in Dulbecco's modified Eagle's medium (DMEM) with 10% fetal bovine serum (FBS), and L02 cells were grown in RPMI 1640 medium with 10% FBS at 37°C in 5% CO_2_. 293-GFP-LC3 cell line and LC3-GFP plasmid were gifts from Professor Liu Wei in Zhejiang University (49). Transient transfection for knockdown was carried out with Lipofectamine RNAiMAX (Invitrogen, 13778-015) according to the manufacturer's instructions. Transient transfection for overexpression was carried out with Lip3000 (Invitrogen, L3000015).

### Reagents, Antibodies, and Plasmids

Baf-A1 (s1413), Torin 1 (s2827), 3-MA (s2767), and Chloroquine diphosphate(CQ, s4157)were purchased from Selleck. The following antibodies were used: anti-LC3 (Sigma, L7645), anti-ACTB/β-actin (Cell Signaling, A5316), anti-phospho-p70 S6 kinase (Thr389) (Cell Signaling, 9234), anti-phospho-4E-BP1 (Thr37/46) (Cell Signaling, 2855), anti-SQSTM1/p62 (MBL PM045), anti-DHX15 (Abcam, ab70454), anti-Ki67 (Cell Signaling, 9449), anti-ULK1 (Cell Signaling, 8054), anti-phospho-ULK1(Ser757) (Cell Signaling, 14202), anti -p70 S6 kinase (Cell Signaling, 2708), anti-4E-BP1 (Cell Signaling, 9452) and CoraLite488–conjugated Affinipure goat anti-mouse IgG (H+L) (Proteintech, SA00013-1). DHX15-Flag was created in the CV702 by standard subcloning. DHX15 plasmid and its negative control were purchased from Genechem (Shanghai, China).

### Protein Extraction and Western Blotting

Ice-cold phosphate-buffered saline (PBS) was used to wash cells three times, and the total proteins were then extracted using extraction reagent (Thermo Fisher, 78501) according to the manufacturer's protocol. After quantification using a BCA Protein Assay Kit (Thermo Fisher, 23227), samples containing equal amounts of protein were loaded onto gels and separated by sodium dodecyl sulfate-polyacrylamide gel electrophoresis (SDS-PAGE). Afterwards, the proteins were transferred to 0.2 μm PVDF membranes and blocked in TBS-T [150 mM NaCl, 10 mM Tris-HCl (pH 7.5), and 0.1% Tween 20] containing 5% bovine serum albumin (BSA) or skim milk powder for 1 h. Then, the corresponding primary antibodies were added, and the membranes were incubated overnight at 4°C. The membranes were incubated with secondary antibodies for 1 h at room temperature. Data are representative western blots of three independent assays.

### RNA Interference

All siRNAs used in this study were purchased from RiBo Biology (Guangzhou, China). The following siRNAs were used: siDHX15 (stB0006121A), 5′-GGGCATTACTTAACTGTGA-3′;

siATG5 (siG10726164423), 5′-GTGAGATATGGTTTGAATA-3″; and a negative control siRNA. Cells were transfected with siRNAs (at a final concentration of 100 nM) using Lipofectamine RNAiMAX reagent. After 48 or 72 h, the cells were analyzed for target expression, autophagy, DHX15 expression, and DHX15 knockdown-mediated changes in cell proliferation.

### Confocal Microscopy

After 48 h of transfection, cells were washed with PBS three times. After treatment with 4% paraformaldehyde (PFA) for 15 min, the cells were washed with PBS three times. The cellular nuclei were counterstained with DAPI. A Carl Zeiss LSM 880 fluorescence microscope was used to capture images showing the cellular localization of LC3B puncta. All of the puncta were analyzed with ImageJ software. The numbers of fluorescent LC3B puncta were determined by counting in more than 100 cells in triplicate.

### Transmission Electron Microscopy

After 48 h of siRNA transfection, L02 cells were fixed in 2.5% glutaraldehyde in 0.2 M HEPES (pH 7.4) and post-fixed in aqueous 1% OsO_4_ followed by 2% uranyl acetate. After ethanol and propylene oxide dehydration and embedding in Polybed 812 resin, ultrathin (80 nm) sections were post-stained with 2% uranyl acetate followed by 0.3% lead citrate. Sections were imaged using an H-7650 transmission electron microscope (Hitachi Company) at 80 kV. For autophagic vacuole quantification, 10 micrographs at a primary magnification of 25,000× were obtained with systematic random sampling of each sample.

### RNA Extraction and Expression Analysis

TRIzol reagent was used for RNA extraction following the manufacturer's instructions. Purified RNA was converted to cDNA using a reverse transcription kit (Takara), and quantitative PCR (qPCR) was performed on an ABI 7900 real-time PCR system using FAST SYBR Master mix (all from Takara). GAPDH expression was used to normalize gene expression.

### Autophagy-Related Treatments

For Torin 1 treatment, cells were cultured with 250 nM Torin 1 for 6 h. Baf-A1 at 200 nM was used to treat cells for 4 h. 3-MA at 5 mM was used to treat cells for 2 h.

### Immunofluorescence

After 48 h of transfection, cells were washed with PBS three times. After 15 min of treatment with 4% PFA, the cells were washed with PBS three times for 5 min each. Then, the cells were permeabilized in 0.5% Triton X-100 for 20 min at room temperature. After blocking in 3% BSA for 30 min at room temperature, the cells were incubated with primary antibodies diluted in blocking buffer overnight at 4°C. The cells were then incubated with secondary antibodies diluted in blocking buffer at room temperature for 1 h in the dark. PBS was used to wash the cells three times for 5 min each. The nuclei were counterstained with DAPI. The stained cells were observed with an inverted fluorescence light microscope (Carl Zeiss LSM 880). The numbers of fluorescent Ki67-positive puncta were determined by counting in more than 100 cells in triplicate.

### Cell Viability Assay

Cells were counted and seeded in 96-well plates with 100 μl of cell suspension per well. After transfection for 48 h, Cell Counting Kit-8 (CCK-8) solution was added to each well. The 96-well plates containing CCK-8 solution were incubated for 1–3 h. The absorbance (450 nm) of the samples was measured, and the relative cell viability was calculated.

### Colony Formation Assay

HepG2 cells were seeded at a density of 1,000 cells/well in six-well plates. The cells were transfected with each siRNA and incubated in DMEM with 10% FBS at 37°C in 5% CO_2_ for 12 days. After 15 min of treatment with 4% PFA, the cells were washed with PBS three times. Then, the colonies were stained with Coomassie brilliant blue for 35 min. Finally, the staining solution was washed away with distilled water. The total number of colonies was determined.

### Human Samples

Human liver cancer tissues were taken from the First Affiliated Hospital, Zhejiang University School of Medicine. Twenty pairs of tumor liver tissues and their adjacent non-tumor tissues were collected from HCC patients after surgical resection. All the diagnoses of 20 patients were based on clinical and pathological examination. The clinicopathological characteristics of the 20 patients are summarized in Supplementary Table 1. All the human tissues were taken with written informed consent and with the approval of the Medical Ethical Committee of the First Affiliated Hospital, Zhejiang University School of Medicine.

### Immunohistochemistry

*In vivo* DHX15 expression was detected by immunohistochemistry using 20 pairs of cancer tissues and adjacent normal-tissues (santa cruz, sc-271686). Immunohistochemistry was performed according to standard protocols, the sections were scanned, and the images were then digitalized. The integrated optical density of DHX15 was calculated using Image-J. The clinicopathological characteristics of the 20 patients are summarized in Supplementary Table 1.

### Statistical Analysis

All measurements were performed at least in triplicate. The data are presented as the mean ± SEM or SD. Student's *t-*test was used to evaluate differences between groups. *P* < 0.05 indicated statistical significance.

## Data Availability Statement

The original contributions presented in the study are included in the article/Supplementary Material, further inquiries can be directed to the corresponding author/s.

## Ethics Statement

The studies involving human participants were reviewed and approved by the Medical Ethical Committee of the First Affiliated Hospital, Zhejiang University School of Medicine. The patients provided their written informed consent to participate in this study.

## Author Contributions

MZha wrote the manuscript and prepared the figures. LY collected and analyzed the human samples. JY and RW amended the grammar of the manuscript. LZ provided some technical advice. ZY, MZhe, and ZC provided expert comments and edits. All authors read and approved the final manuscript.

## Conflict of Interest

The authors declare that the research was conducted in the absence of any commercial or financial relationships that could be construed as a potential conflict of interest.

## Abbreviations

DHX15: DEAH box helicase 15
mTORC1: mammalian target of rapamycin complex 1
MAP1LC3 (LC3): microtubule-associated protein 1 light chain 3 beta
p62: Sequestosome-1
AMPK: AMP-activated protein kinase
70S6K: 70 S6 kinase 1
4EBP1: eukaryotic translation initiation factor 4E-binding protein 1
3-MA: 3-methyladenine
GFP: green fluorescent protein
Ki67: marker of proliferation Ki67
ATG: autophagy-associated gene
Baf-A1: bafilomycin A1
CQ: Chloroquine diphosphate.
