# Supplementary material for: DHX15 Inhibits Autophagy and the Proliferation of Hepatoma Cells
Source: Front Med (Lausanne). 2021 Feb 11;7:591736. doi: 10.3389/fmed.2020.591736 (PMC7904900; doi:10.3389/fmed.2020.591736)

**
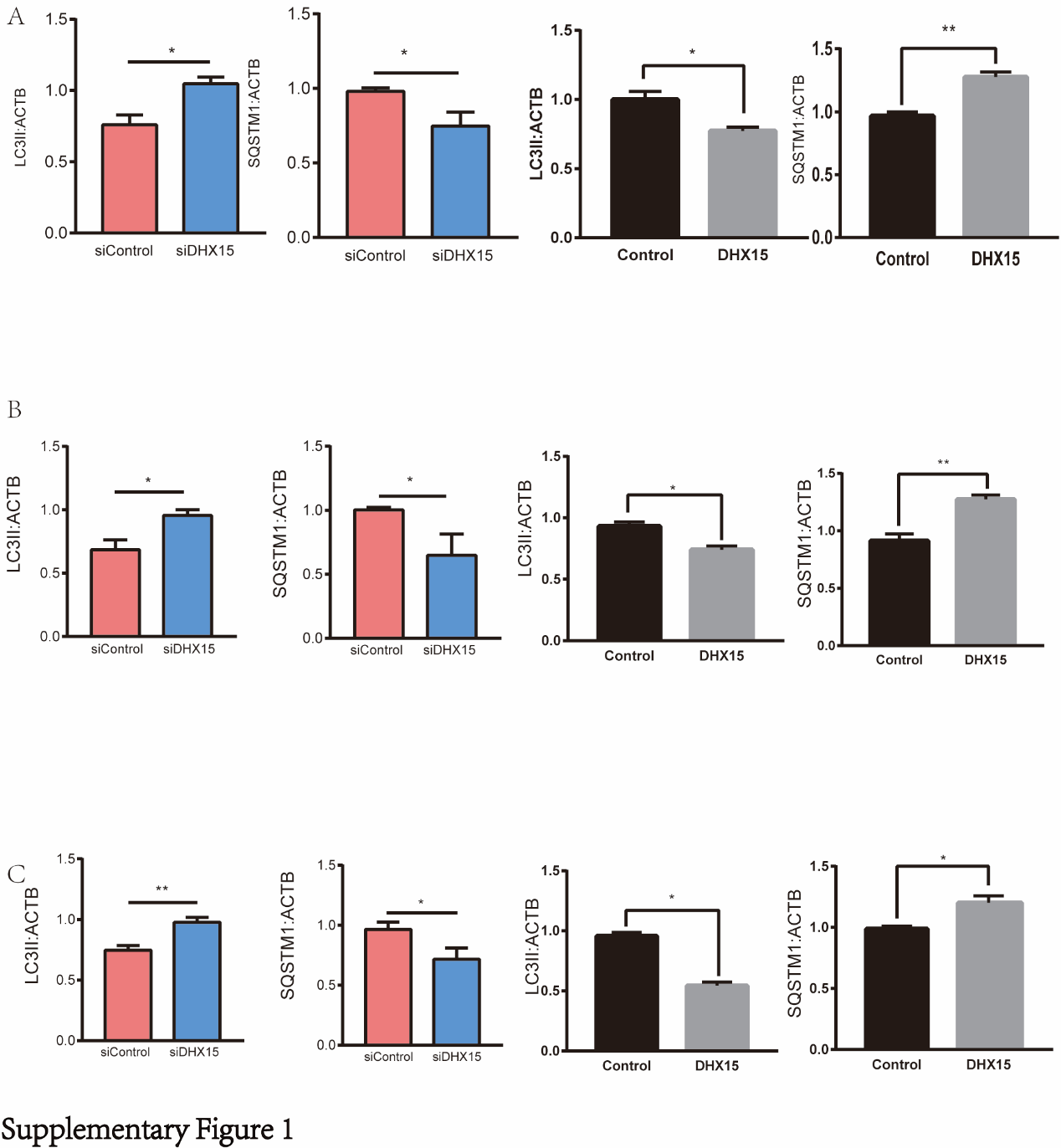
**

**Supplementary Figure 1** Downregulation of endogenous DHX15 induces autophagy

1. Western blot analysis was performed to detect autophagy level after treatment of L02 cells with siDHX15 or DHX15 plasmid for 48h.β-Actin was used as a loading control.*P<0.05. Data obtained from three independent experiments.(B) Huh7 cells transiently transfected with siDHX15 or DHX15 plasmid for 48h to detected autophagy level and harvested for western blotting. β-Actin was used as a loading control. *P<0.05. Data obtained from three independent experiments. (C)Western blot analysis was performed to detect autophagy level after treatment of HepG2 cells with siDHX15 or DHX15 plasmid for 48h. β-Actin was used as a loading control.^*^P<0.05,^**^P<0.01. Data obtained from three independent experiments.


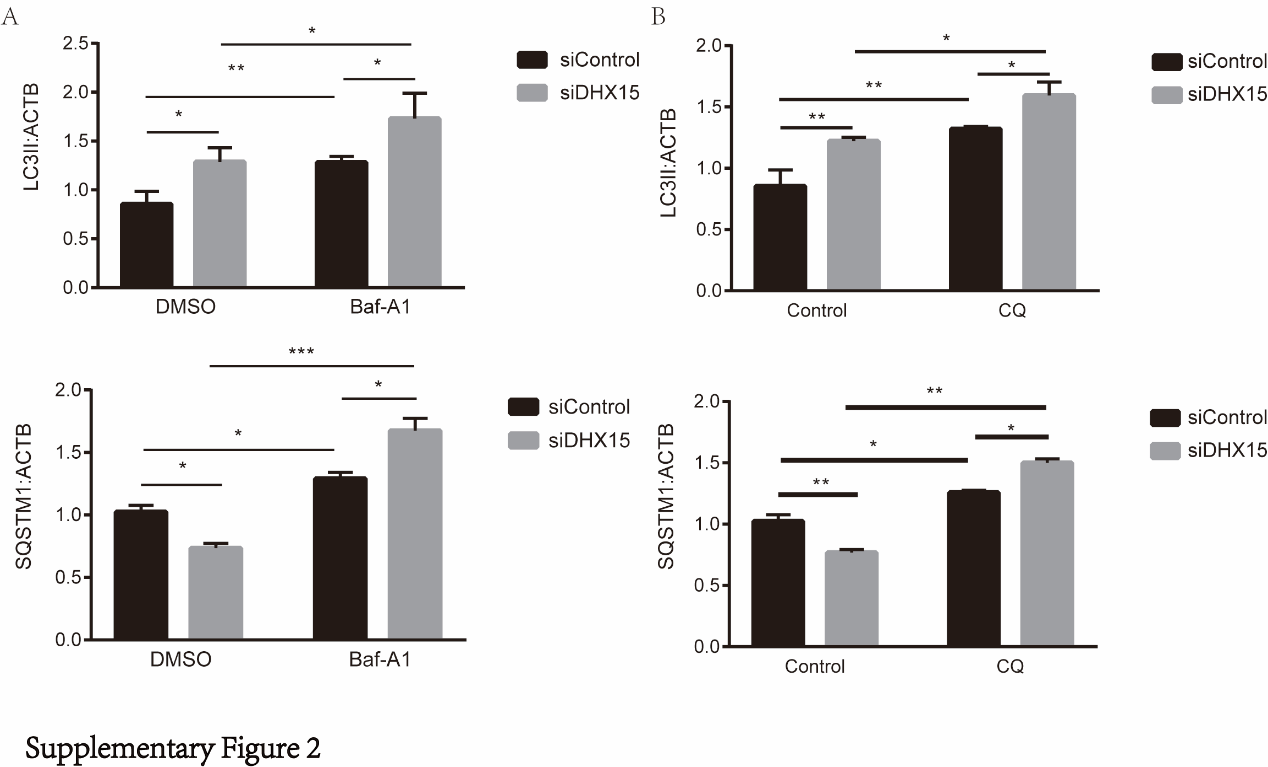


**Supplementary Figure 2** Knockdown of DHX15 promotes the formation of autophagosomes.

(A)Huh7 cells transiently transfected with siDHX15 or siControl for 48h with or without Baf-A1 (200nM) 4h to detected autophagy level and harvested for western blotting. β-Actin was used as a loading control. ^*^P<0.05, ^**^P<0.01,^***^P<0.001. Data obtained from three independent experiments. (B)Huh7 cells transiently transfected with siDHX15 or siControl for 48h with or without CQ(20uM) 24h to detected autophagy level and harvested for western blotting. β-Actin was used as a loading control. ^*^P<0.05, ^**^P<0.01 ,^***^P<0.001 . Data obtained from three independent experiments.

**
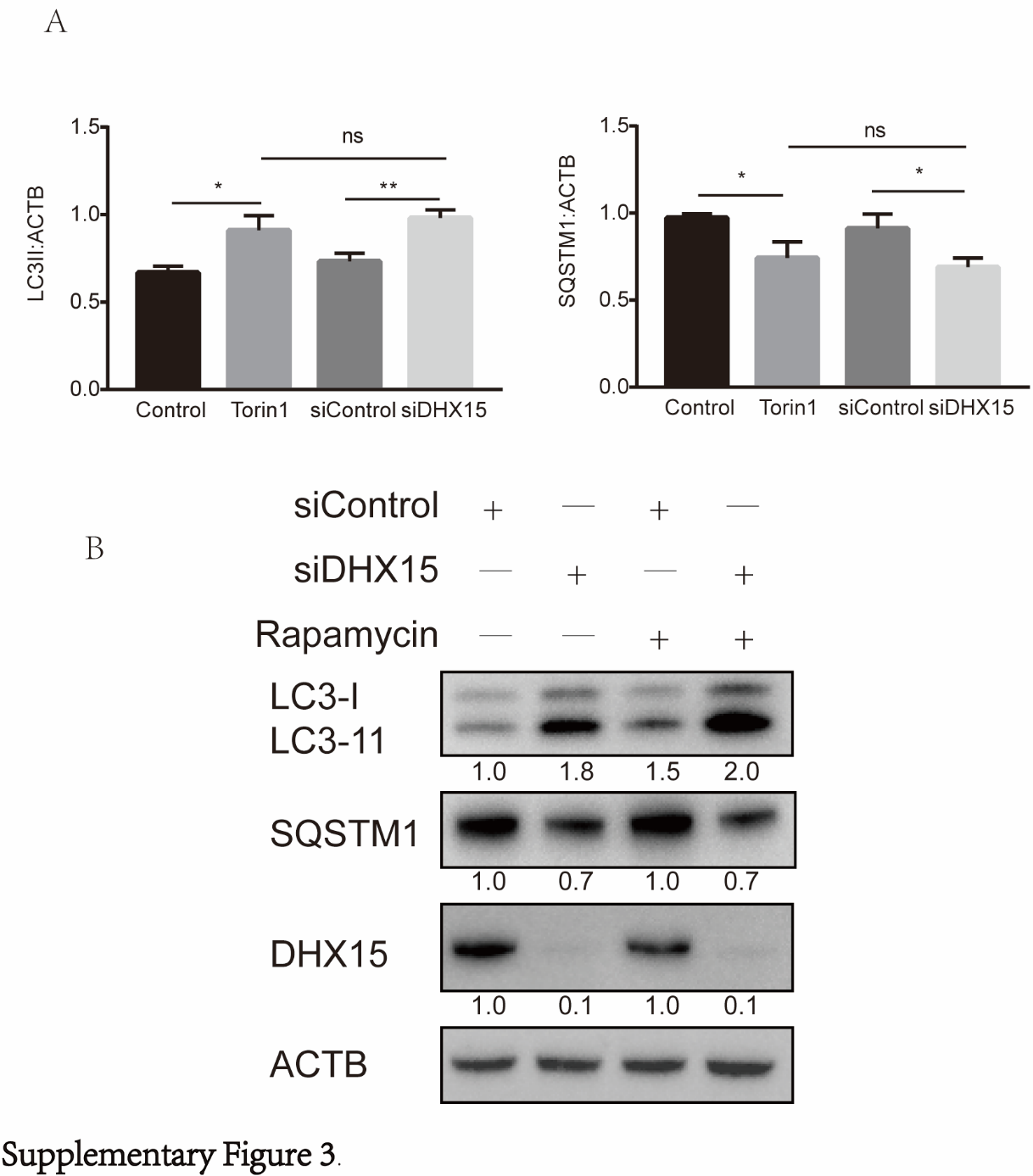
**

**Supplementary Figure 3** DHX15 regulates autophagy in a manner associated with mTORC1 activation. (A) Western blot analysis was performed to detect autophagy level after treatment of HepG2 cells with Torin1 250nM 6h or siDHX15 48h.*P<0.05,**P<0.01. Data obtained from three independent experiments. (B) HepG2 cells transiently transfected with siDHX15 or siControl were treated with 200 nM rapamycin for 2 h and harvested for western blotting. Treatment with DMSO was used as a negative control. β-Actin was used as a loading control. ^*^P<0.05.


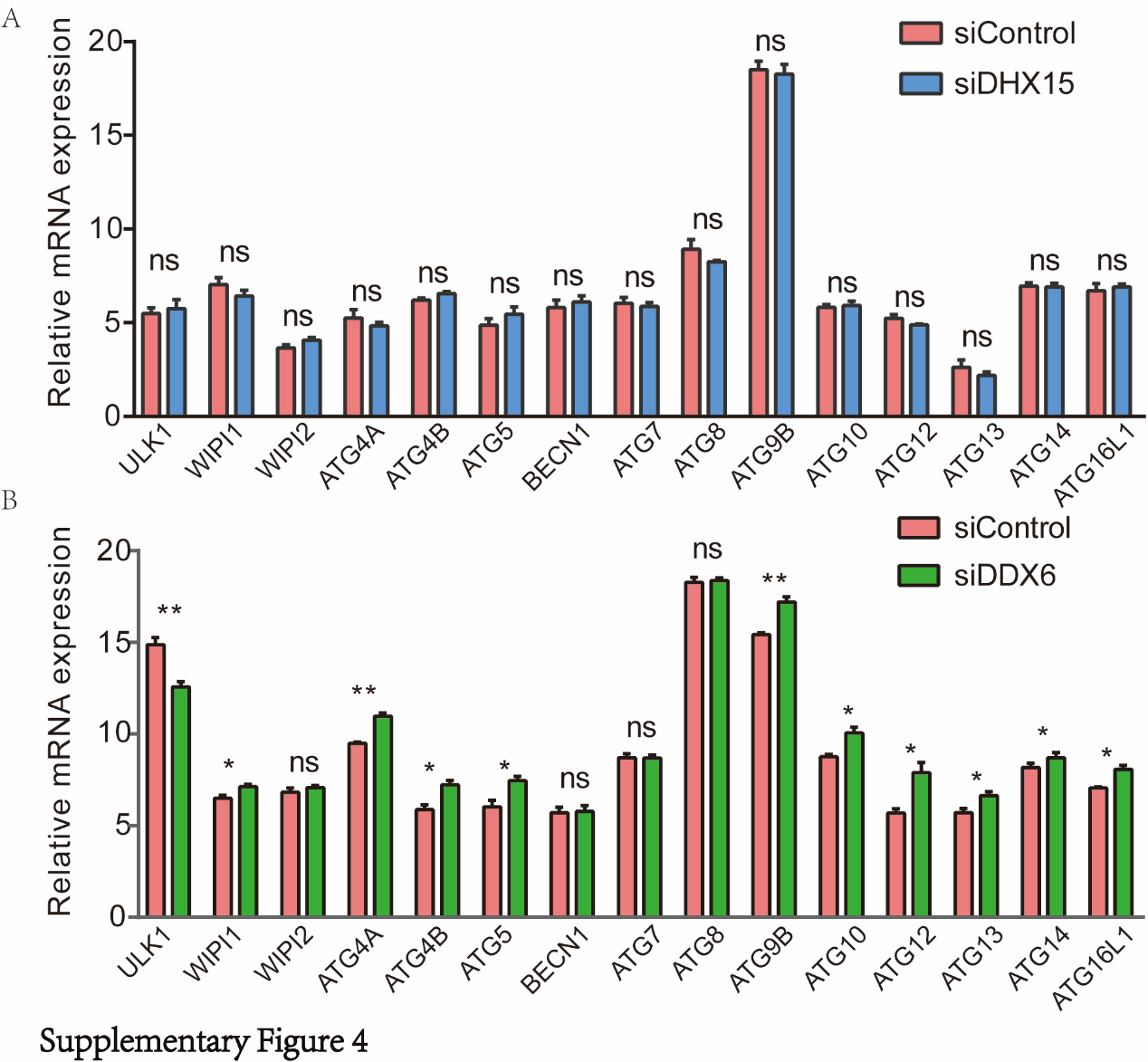


**Supplementary Figure 4** DHX15 does not affect the mRNA expression of autophagy-related genes. (A) The mRNA levels of autophagy-related genes in HepG2 cells transiently transfected with DHX15 siRNA (siDHX15) or scrambled control siRNA (siControl) were detected by real-time qPCR. The data are presented as the mean ± SEM (n = 3). (B) Autophagic gene mRNA levels were detected in HepG2 cells in which endogenous DDX6 has been knocked down. The data are presented as the mean ± SEM (n = 3).

**
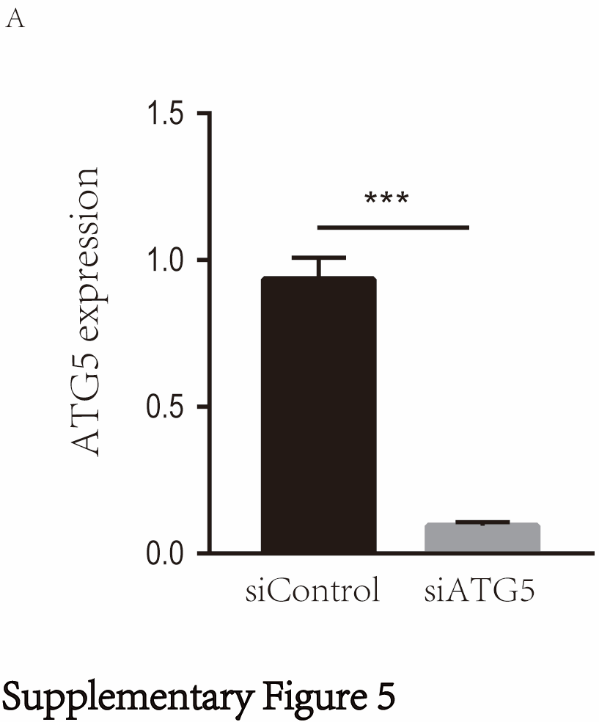
**

**Supplementary Figure 5** DHX15 inhibits the proliferation of hepatoma cells in an autophagy-dependent manner. (A) HepG2 cells were transiently transfected with siATG15 or siControl for 48h to detected ATG5 mRNA level. ^***^P<0.001. Data obtained from three independent experiments.


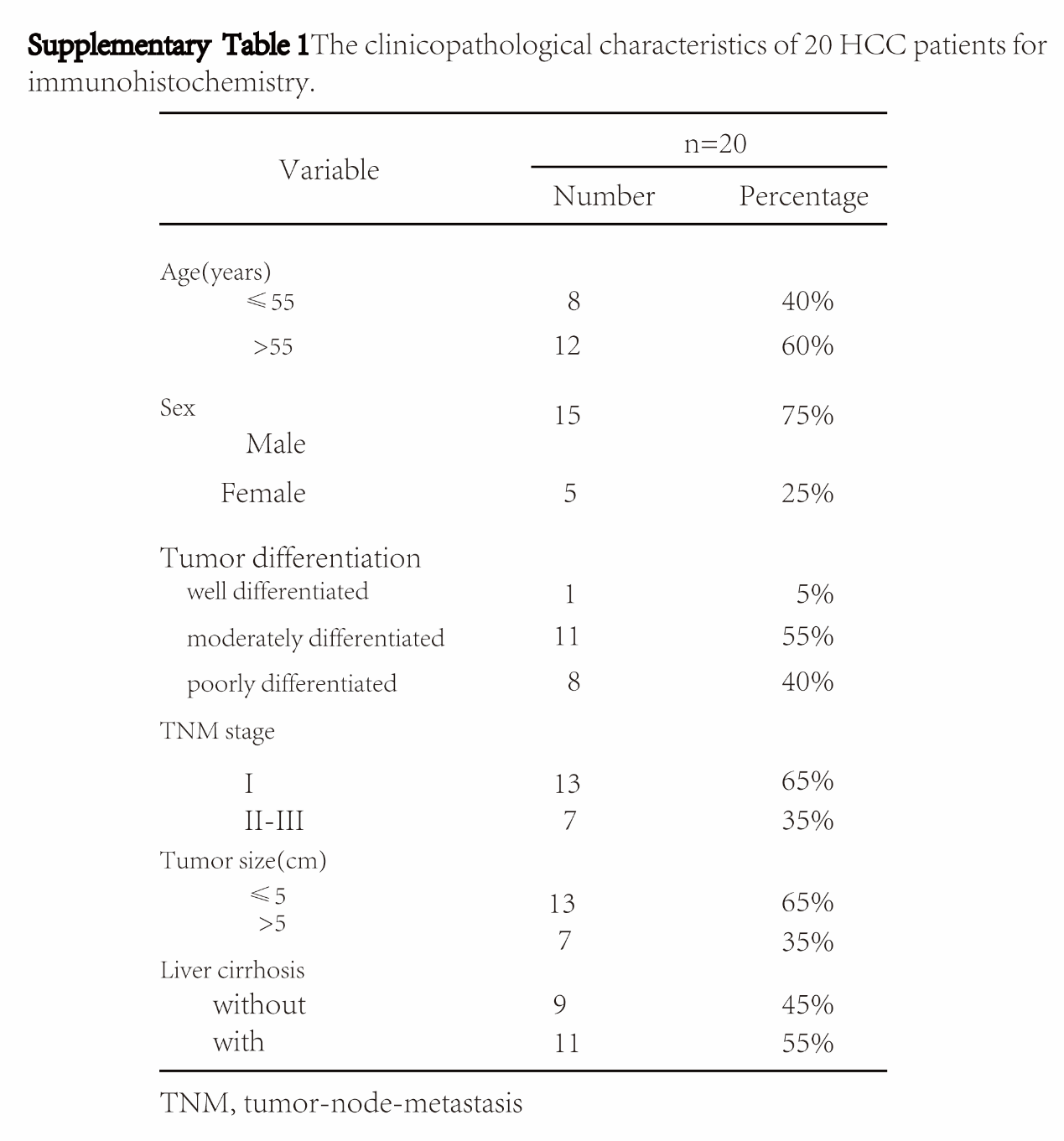

Supplement: Supplementary file 1 [file Data_Sheet_1.docx]
